# Maternal immune markers in serum during gestation and in breast milk and the risk of asthma-like symptoms at ages 6 and 12 months: a longitudinal study

**DOI:** 10.1186/1710-1492-8-11

**Published:** 2012-07-17

**Authors:** Nelís Soto-Ramírez, Wilfried Karmaus, Mitra Yousefi, Hongmei Zhang, Jihong Liu, Venugopal Gangur

**Affiliations:** 1 Epidemiology and Biostatistics Department, Norman J Arnold School of Public Health, University of South Carolina, 800 Sumter Street, Columbia, SC, 29208, USA; 2 Food Allergy and Immunology, Department of Food Science and Human Nutrition, Michigan State University, 302B G.M. Trout FSHN Building, East Lansing, MI, 48824-1224, USA

**Keywords:** Breast milk, Maternal serum, Immune markers, Cytokines, Chemokines, Asthma-like symptoms, Children, Longitudinal study

## Abstract

**Background:**

The role of breast milk on the risk of childhood asthma is in dispute. The aim of this prospective study is to determine the relationship of immune markers in maternal serum during gestation and breast milk to asthma-like symptoms (AS) in infancy.

**Methods:**

Pregnant women were recruited in Columbia and Charleston, South Carolina. Blood (median: three weeks before delivery) and breast milk (three weeks after delivery) samples were collected. Concentrations of interferon (IFN)-γ, IFN gamma-induced protein 10 (IP-10 or CXCL10), CCL11, interleukin (IL) 1β, IL-4, IL-5, IL-6, CXCL8, IL-10, IL-12(p70), IL-13, transforming growth factor (TGF)-β1, and immunoglobulin (Ig) A in both maternal serum and milk whey were determined via immunoassays. Asthma-like symptoms (AS) of the infant were ascertained at 6 and 12 months, respectively. Generalized estimating equations assessed relative risks (RRs) of immune markers for repeated measurements of AS, considering intra-individual correlations and adjusting for confounders. To provide comparable risk estimates, quartiles of the immune markers were used, except for IL-5 in whey and IgA in serum, which were dichotomized.

**Results:**

Of 178 women, 161 provided blood and 115 breast milk samples. IL-12(p70), IL-4, IL-10, IL-1β, and CCL11 in serum and in whey were not further considered for the statistical analyses since the proportion of non-detectable values was high. Most immune markers in serum and milk whey were moderately or highly correlated; however, IgA was negatively correlated. Infants in the highest quartile of IL-13 in both serum and whey were at a higher risk of AS (RR = 3.02 and 4.18; respectively) compared to infants in the first quartile. High levels of IL-5 in serum and whey was also identified as a risk. In addition, increased secretory IgA and TGF-β1 in breast milk reduced the risks of AS.

**Conclusions:**

Maternal serum and whey levels of IL-5 and IL-13 are risk markers for AS; whey IgA and TGF-β1 seem to be protective. Only focusing on breast milk portend that milk cytokines IL-5 and IL-13 have adverse effects. However, similar immune exposures during late gestation and via milk suggest that both may enhance AS among infants.

## Background

Allergic disorders such as eczema, asthma, and hay fever are among the most common chronic diseases during childhood [1]. In the United States, more than 10 million children under 18 years of age have ever been diagnosed with asthma (14%); almost 7 million children have ongoing asthma (10%) [2]. The socioeconomic burden of asthma is striking [3]. Despite considerable progress in research, an understanding of the etiology of childhood asthma remains incomplete. Breast milk contains a variety of immune molecules such as immunoglobulins, cytokines, chemokines, and growth factors, which are believed to protect the infant from common pathogens. There is a consensus of a protective effect of breastfeeding on lowering the risk or severity of respiratory infections [4,5]. However, for asthma, the question of whether breastfeeding is protective or a risk remains controversial. Some studies report that exclusive breastfeeding is associated with a reduced risk [6-14], while others report an increased risk of asthma [15,16] or no association [17-19].

Breast milk related factors that are responsible for a potential association with asthma risk have not yet been clearly identified. Immune markers, particularly, the T helper (Th)1/Th2/Th3/T-regulatory pathways are thought to be critical players. Th1 cells are responsible for cell-mediated immunity and phagocyte-dependent protective responses, while Th2 cells are associated with antibody production, eosinophil activation, and inhibition of several macrophage functions [20]. Studies reported that cytokines and chemokines related to eosinophil activation (such as IL-5, CCL11) and production of immunoglobulin E (such as IL-4, IL-13) are responsible for allergies in children [21,22].

Past studies have focused on breast milk and asthma-like symptoms (AS) in infancy but ignored whether maternal immune response may influence the fetal immune system. The aim of the longitudinal study is to determine whether different proteins in breast milk whey and maternal serum before delivery are linked to AS in infants at ages 6 and 12 months. We focused on levels of Type-1/pro-inflammatory cytokines/chemokines (interferon (IFN)-γ, interferon gamma-induced protein 10 (IP-10 or CXCL10), interleukin (IL)-1β, IL-6, IL-12(p70), CXCL8 (IL-8)), Type-2/pro-allergic cytokines/chemokines (IL-4, IL-5, IL-13, and CCL11 (eotaxin)), T-regulatory/anti-inflammatory cytokines (transforming growth factor (TGF)-β1 and IL-10), and the secretory immunoglobulin A (IgA).

## Methods

This investigation was derived from two ongoing longitudinal studies, which were approved by the University of South Carolina Institutional Review Boards, the Medical University of South Carolina, and the Palmetto Health Institutional Review Boards for Human Subjects. All participants signed a written consent form either in English or Spanish.

### Participants

Expecting mothers in the second trimester were enrolled between April 2008 and January 2010 in Columbia and Charleston, South Carolina, in prenatal clinics and prenatal classes. Trained staff recruiters invited the expecting mothers to participate in the study at the prenatal classes and by research nurses performed recruitment tasks at the prenatal clinics. Eligibility criteria included: (1) aged 18 years or older, (2) no chronic illness (diabetes, thyroid or adrenal disorders, or chronic infections), (3) planned to stay in the area for at least nine months, and (4) willingness to provide a breast milk sample two weeks after delivery.

### Questionnaires and potential confounders

Women took part in four telephone or in-person interviews: a core demographic and baseline interview conducted before delivery, and three interviews at 2 weeks, and 6 and 12 months after delivery, respectively. At the baseline interview, information was obtained about women’s socio-demographic and medical factors including race (African American, Caucasian or other), maternal age, maternal and household smoking during pregnancy period (yes, no), education level (less than high school, some college, college graduate, or graduate school), and maternal history of allergy (eczema, asthma, and rhinitis). The maternal history of asthma, wheezing and whistling in the chest, and eczema was obtained by asking: “Have you ever had asthma?” and “Have you ever had wheezing or whistling in the chest at any time in the past?” The next two questions were grouped to define rhinitis: “Have you ever had hay fever?” and “Have you ever suffered - in the absence of a cold - from an itchy stuffy or runny nose and/or swollen, itchy eyes?” The question “Have you ever had an itchy rash, which was coming and going for at least six months?” was used as an indicator of eczema.

The second interview was conducted 2 to 4 weeks after delivery. It provided information on delivery date, gestational age (weeks), mode of delivery (spontaneous vaginal delivery, after induction vaginal delivery, Cesarean section), gender of the offspring, maternal and child health status, maternal and household smoking during pregnancy period (yes, no), maternal use of antibiotics during pregnancy, and history of vaginal infections/pelvic conditions during pregnancy. The birth date was utilized to assign the season at birth based on pollen seasons in South Carolina [fall (September, October, November) and spring (March, April, May)]. The non-pollen seasons included winter (December, January, and February) and summer (June, July, and August).

The third and fourth interviews were based on the International Study of Asthma and Allergies in Childhood (ISAAC) questionnaire [23] and ascertained asthma-like symptoms at ages 6 and 12 months. The recall period was restricted to either the previous 6 or 12 months. In the case that a 6 month interview was performed at 7 months after delivery, the mother was still asked to report on only the period of time before the child turned age 6 months. Additionally, the interview collected information on duration of breastfeeding (weeks) as well as information on smoking in the house in the last 6 months (yes, no). At 6 and 12 months, the following question was asked for ascertaining the different respiratory infections of the child in the last 6 months: “In the last 6 months, was a doctor’s diagnosis made in your child of (yes, no): (1) pneumonia, (2) wheezy bronchitis, (3) infectious bronchitis, or (4) middle-ear infection.”

### Blood and breast milk collection and preparation

Women were asked to provide one blood sample before delivery and one breast milk sample about two weeks after delivery. Ten milliliters of blood were taken by venipuncture from each woman in the last trimester of pregnancy (range: 0 to 13 weeks before delivery, median: 3 weeks before delivery). All serum samples were collected in sterile tubes (BD Vacutainer ®, 10 mL), and centrifuged within one hour of collection at 3,500 revolutions per minute (rpm) for 10 minutes (min) at 4 degrees Celsius (°C). The separated serum samples were stored at −20°C then after 24 hours were transferred to −80°C where the samples were stored until needed for analysis.

Each participant expressed approximately 15 mL of breast milk, using an electric breast pump provided by the study, on average three weeks after delivery (range 1–8 weeks). All women followed a detailed breast milk collection protocol. Women were asked to collect breast milk during the morning and after putting the baby to the breast. The nipple and surrounding breast area were cleaned with sterile wipes prior to breast milk collection. All breast milk samples were collected in sterile plastic bottles (Medela 80 ml [2.7 oz]). Research staffs picked the breast milk sample up at the participant residence. Within one hour of collection, all samples were transferred to sterile centrifuges tubes and spun at 2,900 rpm for 10 minutes at 4°C. Fat was removed; centrifugation and fat removal steps were repeated until all fat was taken out. Finally, the cell pellet was removed. The isolated whey and serum were aliquoted and stored in a −80°C freezer until preparation for immunoassays.

### Immuno-assay protocols

The concentrations of IL-1β, IL-4, IL-5, IL-6, CXCL8, IL-10, IL-12(p70), IL-13, CXCL10, CCL11, and IFN-γ in both serum and whey were assayed using the Bioplex Protein Array system (BioRad, Bio-Rad Laboratories, Inc., Hercules, CA). This multiplexes system allows for the assessment of several immune markers in the same Biorad custom-made bioplex pro human cytokine, chemokine, and growth factor multiplex plate. ELISAs (enzyme-linked immunosorbent assay) were used to determine concentrations of IgA (Immunology Consultants Laboratory) and TGF-β1 (R&D Systems). To activate latent TGF-β1 to immuno-reactive TGF-β1 detectable by the Quantikine TGF-β1 immunoassay, we followed the manufacture procedure [24]. All assays were conducted according to the manufacturer’s kit instructions [24-26]. Each sample, including standards and the blank, was assayed in duplicate. A total of 15 multiplexes were performed to determine the concentration of the immune markers in serum and whey. For each plate we determined the limit of detection (LOD) by multiplying the standard deviation of the blank by three. Those samples that had concentrations below the detection limit were assigned a value corresponding to half the LOD.

### Outcome variable

Asthma like-symptoms (AS) in infants were ascertained prospectively at ages 6 and 12 months by the ISAAC questionnaire. AS included the following items [options: yes, no, if not stated otherwise]: 1) has your child had wheezing or whistling in the chest in the last 6 months? 2) how many attacks of wheezing has your child had in the last 6 months? (options: more than twelve; three to twelve; one to two; none – dichotomized to one or more vs. none) 3) has your child been unwell when breathing in or making noise when doing so in the last 6 months; and 4) has your child suffered from a shortness of breath in the last 6 months? Any positive answer to one of these four questions was considered as presence of AS.

### Exposures

The main exposures were immune markers levels in maternal serum before delivery and in whey. The immune markers included in the analyses were the following: Type-1/pro-inflammatory cytokines/chemokines (IFN-γ, CXCL10, IL-6, and CXCL8), Type-2/pro-allergic cytokines/chemokines (IL-5 and IL-13), T-regulatory/anti-inflammatory cytokine (TGF-β1) and IgA levels. The concentrations were recorded in pg/mL, except for IgA, which was measured in mg/mL. Due to their distributions, we categorized all immune markers into quartiles except for IL-5 in whey and IgA in serum, which were dichotomized.

### Data analysis

To investigate the association of serum and whey immune markers, intra-class and Spearman correlation coefficients were estimated. The following confounders were controlled in the statistical analyses. Maternal characteristics included race, age at pregnancy, smoking during pregnancy, household cigarette use at ages 6 and 12 months, maternal history of asthma, eczema, and rhinitis, consumption of antibiotics during pregnancy, and vaginal infections/pelvic conditions during pregnancy. Offspring characteristics comprised of gender, any respiratory infections at ages 6 and 12 months, and season of birth. Indicators of respiratory infections during infancy included a doctor’s diagnosis of pneumonia, wheezy bronchitis, infectious bronchitis, and middle-ear infection. Gestational age, maternal education, pet exposure, preconception body mass index, and mode of delivery were removed from the models because they were not confounding.

Log-linear regression was used to test whether immune markers were associated with AS at age 6 months and with ever AS in the first year of life (supplemental material). Generalized estimating equations were applied to predict repeated occurrence of AS in infants at ages 6 and 12 months. Adjusting for within-participant effects using the regular maximum likelihood method, we started with an unstructured covariance matrix, which requires the least amount of constraints. Other covariance matrices, including compound symmetry and autoregressive, were considered and most of the models presented the same QIC goodness of fit statistic [27].

A total of 16 adjusted models (8 for each serum and whey markers) were run to analyze the effect of immune markers on AS. Initially, we considered analyzing serum and whey immune markers in one model; however, this led to collinearity problems [28], since most of the immune markers presented correlations above 0.5 (e.g., CXCL10 and CXCL8 in whey r = 0.68). In addition, the possibility that whey’s immune markers are in the ‘causal pathway’ between immune markers in serum and AS cannot be tested with simple regression models since these models do not allow for intervening variables. Controlling for an intervening variable as a confounder would split the initial association between the risk factor and the outcome into two associations, destroying the ‘causal pathway’. For these reasons, we assessed all immune markers in serum and whey separately. We detected that the immune markers had non-linear relations with AS. We tested for linearity by cross-tabulating groups with increasing immune marker levels with the outcome. We therefore analyzed all the immune markers (with the exception of IL-5 in whey and IgA in serum) by comparing risk ratio (RR) estimates for AS, contrasting the upper quartiles with the lowest quartile (low concentration) as reference group. IL-5 in whey and IgA in serum were dichotomized (high versus low) by using the median as a cut off since their distributions were skewed and small proportion of individuals with AS information in the lowest quartile (reference group). We estimated the 95% confidence interval (CI) and also presented p-values to identify associations that survive penalizing for multiple testing.

We are interested in understanding whether both immune markers in maternal serum and whey are primarily associated with AS in offspring. Given the time sequence of serum preceding whey, this could shed light on whether pre- or post-natal cytokine levels are the culprit for asthma-like symptoms in infancy. To address this question using one correlated cytokine variable, we created high and low concentration combinations of IL-13 and IL-5 using the median as a cut-off point (these cytokines were chosen because their individual concentrations were significantly associated with AS). The resulting levels were categorized as follows: 1) high levels in serum/high levels in whey, 2) low levels in serum/high levels in whey, and 3) low levels in serum/low levels in whey. Since the combination “high levels in serum/low levels in whey” had two or less observations, we did not consider this combination for the analysis.

All confounders were simultaneously entered as indicator variables into the generalized mixed models. A backward elimination process was used to retain confounders in the final model: confounders were those which changed the effect of the main association by 10% or more when omitting this factor from the model. To address confounding due to respiratory infections during infancy, we examined any of the four markers of respiratory infections and wheezy bronchitis as confounders. In addition, we also ran models excluding infants who had both wheezy bronchitis and AS. To adjust for multiple testing, we applied a separate false discovery rate (p = 0.05) for serum and whey immune markers [29]. All statistical analyses were performed using SAS version 9.2 (SAS Institute Inc., Cary, NC, USA).

## Results

Of 178 women who participated, 161 provided maternal blood and 115 breast milk samples (98 provided both prenatal blood sample and breast milk sample). Most of the participants were Caucasian (73.5%) and highly educated. A history of maternal eczema was reported by 9.1%, a history of asthma by 27.4%, and a history of rhinitis by 48.2% (Table 1). About 5% reported smoking during pregnancy, and 45% had a vaginal infections/pelvic conditions during pregnancy.

**Table 1:** Characteristics of the participants

**Variables**	**Pre-delivery**	**Breast milk**	**All samples**
	**serum**	**n = 115**	**N = 178**
	**n = 161**	**n (%)**	**N (%)**
	**n (%)**		
***Maternal race***			
African American (AA)	38 (25.5)	20 (17.5)	44 (26.5)
Caucasian or Other	111 (74.5)	94 (82.5)	122 (73.5)
***Maternal education***			
Less than high school	16 (10.8)	9 (7.9)	20 (12.1)
Some college	37 (24.8)	25 (21.9)	41 (24.7)
College graduate	48 (32.2)	36 (31.6)	52 (31.3)
Graduate school	48 (32.2)	44 (38.6)	53 (31.9)
***Mode of delivery***			
Spontaneous vaginal delivery	54 (40.3)	47 (41.6)	61 (40.7)
After induction vaginal delivery	49 (36.6)	38 (33.6)	56 (37.3)
Cesarean section	31 (23.1)	28 (24.8)	33 (22.0)
***Sex of the infant***			
Male	70 (50.4)	55 (48.7)	79 (50.9)
Female	69 (49.6)	58 (51.3)	76 (49.1)
***Maternal smoking during pregnancy***			
Non-smoker	127 (94.8)	105 (93.7)	141 (94.6)
Smoker	7 (5.2)	7 (6.3)	8 (5.4)
***Maternal history of allergy***			
Asthma	42 (28.6)	32 (28.1)	45 (27.4)
Rhinitis	68 (46.3)	60 (52.6)	79 (48.2)
Eczema	13 (8.8)	10 (8.8)	15 (9.1)
***Season of child’s birth***			
Fall	32 (22.7)	23 (20.4)	34 (21.7)
Spring	32 (22.7)	27 (23.9)	34 (21.7)
Summer	30 (21.3)	26 (23.0)	37 (23.6)
Winter	47 (33.3)	37 (32.7)	52 (33.1)
***Gestational use of antibiotics***	29 (18.0)	32 (27.8)	34 (19.1)
Vaginal infections/pelvic conditions during pregnancy*			
Yes	60 (44.8)	49 (43.4)	67 (44.7)
No	74 (55.2)	64 (56.6)	83 (55.3)
	**Mean (n; 5%, 95%)**		
***Maternal age during pregnancy***	29.2	30.9	29.2
	(143; 19.4, 38.9)	(111; 21.7, 38.9)	(158; 19.4, 38.9)
***Gestational age (weeks)***	39.0	39.0	39.0
	(133; 36.0, 41.0)	(112; 36.0, 41.0)	(149; 36.0, 41.0)

* Including urinary tract infection, vaginitis or vaginosis, genital warts, genital herpes, gonorrhea, syphilis, chlamydia, trichomoniasis, yeast infection, and group B *Streptococcus* infections.

Proportions of asthma-like symptoms were 34.8% and 32.4% at ages 6 and 12 months, respectively (Table 2). About 24% (age 6 months) and 49% (age 12 months) of children had respiratory infections. Approximately 9% of infants at age 6 months and 17% at age 12 months presented both AS and any type of respiratory infection (data not shown). Four out of 137 infants at age 6 months and 5 out of 102 infants at age 12 months had both AS and wheezy bronchitis. At ages 6 months, 8.0% (11/137) of the infants and 14.7% (15/102) at 12 months had both mid ear infection and AS. Infectious bronchitis was present in approximately 1% (6 months) and 2% (12 months) of the infants.

**Table 2:** Prevalence of asthma-like symptoms and respiratory infections at ages 6 and/or 12 months (N = 140)

**Asthma-like symptoms (% (n/total))**	**6 months**	**12 months**	**6 or 12 months**	**6 and 12 months**
Wheezing	15.2 (21/138)	13.7 (14/102)	20.7 (29/140)	4.3 (6/140)
One or more wheezing attacks	31.8 (35/110)	26.9 (21/78)	39.0 (48/123)	6.5 (8/123)
Felt unwell and made noise when breathing	13.1 (18/137)	15.7 (16/102)	21.4 (30/140)	2.9 (4/140)
Shortness of breath	2.2 (3/136)	4.9 (5/102)	5.8 (8/139)	0 (0/139)
***Combined asthma-like symptoms***				
Any asthma-like symptom	34.8 (48/138)	32.4 (33/102)	47.9 (67/140)	10.0 (14/140)
**Child respiratory infections**				
Pneumonia	0 (0/137)	3.9 (4/102)	2.9 (4/140)	0 (0/140)
Wheezy bronchitis	3.7 (5/137)	7.8 (8/102)	8.6 (12/140)	0.7 (1/140)
Infectious bronchitis	0.7 (1/137)	1.9 (2/102)	2.1 (3/140)	0 (0/140)
Mid-year infection	22.6 (31/137)	47.1 (48/102)	42.1 (59/140)	14.3 (20/140)
***Combined child respiratory infections***				
Any respiratory infection	24.1 (33/137)	49.0 (50/102)	44.3 (62/140)	15.0 (21/140)

Immune markers IL-12(p70), IL-4, IL-10, IL-1β, and CCL11 in serum and in whey were not considered in the statistical analyses since high proportions of non-detectable values (low or undetectable concentrations) were obtained. The concentrations of CXCL10 and CXCL8 were higher in whey than in serum samples. In addition, most immune markers in maternal serum and whey were moderately to highly intra-correlated (0.35 - 0.66; Table 3), CXCL8 and IgA in serum and whey were negatively correlated (−0.44 and −0.43, respectively).

**Table 3:** Proportion of quantified immune markers, their distribution and correlations between maternal pre-delivery serum and breast milk whey

**Immune markers (concentration in pg/mL for all except IgA in mg/mL)**^†^	**Concentration of immune markers**^**#**^						**Intra-class correlation comparing serum and whey**	**p-value**	**Spearman correlation between serum and whey**	**p-value**
	**Serum (N = 161)**			**Whey (N = 115)**						
	**Proportion of samples with detectable levels % (n)**	**median**	**5%, 95%**	**Proportion of samples with detectable levels % (n)**	**median**	**5%, 95%**				
IL-13	50.0 (80)	1.12	0.02, 9.50	70.4 (81)	0.46	0, 5.31	0.66	<0.0001	0.58	<0.0001
CXCL8	30.5 (39)	2.11	0.42, 8.49	79.8 (67)	5.60	0.40, 265.07	0.02	0.87	−0.44	<0.0001
IL-6	30.5 (39)	2.11	1.06, 27.17	30.9 (26)	1.06	0.21, 20.64	0.36	0.002	0.31	0.007
IL-5	70.3 (90)	1.06	0.03, 2.29	73.8 (62)	0.10	0, 2.12	0.64	<0.0001	0.72	<0.0001
IFN-γ	55.3 (89)	3.89	1.06, 174.22	40.9 (47)	1.59	1.07, 102.53	0.50	<0.0001	0.48	<0.0001
CXCL10	97.5 (157)	169.18	1.91, 2956.89	93.9 (108)	270.25	1.91, 10184.64	0.35	0.002	0.26	0.007
TGF-β1	89.3 (75/84)	23078.87	7.7, 45143.40	93.0 (80/86)	600.88	7.7, 1823.90	0.55	<0.0001	0.31	0.006
IgA	100 (86/86)	5.10	1.98, 8.53	100 (89/89)	4.35	0.13, 2931.48	0.07	0.18	−0.43	<0.0001

^#^A total of 15 multiplexes were performed to determine the concentration of the immune markers in serum and whey. For each plate we determined the limit of detection (LOD) by multiplying the standard deviation of the blank by three. Those samples that had concentrations below the detection limit were assigned a value corresponding to half the LOD.

^†^Immune markers IL-12(p70), IL-4, IL-10, IL-1β, and CCL11 in serum and in whey were not analyzed since high proportion of non-detectable values were obtained.

Regarding the correlation between immune markers in maternal serum, we found that most of the markers were moderately correlated with a spearman correlation coefficient range of 0.36 to 0.80 (supplemental material, Additional file 1: Table E1). The Th2 cytokines in serum, IL-13 and IL-5, had a correlation of 0.77 (p <0.0001), however in whey, IL-13 and IL-5 were not correlated. In addition, in whey IL-13 and IgA in whey were negatively correlated (spearman correlation coefficient = −0.21; p = 0.03; Additional file 1: Table E2).

**Figure 1:**
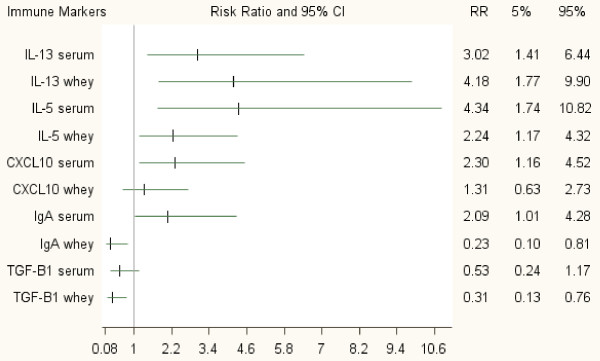
**Adjusted effects of immune markers in maternal serum and in breast milk whey on asthma-like symptoms at ages 6 and 12 months: a repeated measurement analysis.** * Immune markers (except for IL-5 in whey and IgA in serum) were categorized into quartiles using the first quartile (lowest values) as reference. IL-5 in whey and IgA in serum were dichotomized. The risk ratios represent the highest level of the immune marker (4^th^ quartile) compared to the lowest level. IL-5, IL-13, CXCL10, IgA, and TGF-β1 serum and whey were adjusted for child’s sex, maternal age during pregnancy, maternal race, smoking during pregnancy, vaginal infections during pregnancy, maternal history of asthma, eczema, and rhinitis, consumption of antibiotics during pregnancy, season of child’s birth, any respiratory infection during infancy, and household cigarette use at ages 6 and 12 months. ^†^ Gestational age, maternal education, pet exposure, preconception maternal body mass index, and mode of delivery were removed from the models because they were not confounding.

The crude analyses of the repeated measurements of AS indicated that IL-5, IL-13, CXCL10, and IgA in both maternal serum and whey had a significant influence on AS (data not shown). After adjusting for confounders (including any respiratory infections, Table 2), all the aforementioned immune markers in both serum and whey remained significant risk markers for AS (Figure 1; Table 4). For instance, infants in the highest quartile of the serum IL-13 distribution had three times the risk of AS compared to infants in the first quartile. Children exposed to the highest IL-13 level in whey had a significantly higher relative risk for occurrence of AS (RR = 4.18). Regarding IL-5, the risk for AS was 4.34 times higher than the risk in the lowest level in serum, while in whey, children with a high level (dichotomized) of this cytokine had twice the risk of AS compared to infants in the lower level. Regarding IgA, after adjusting for confounders, children exposed to breast milk with the highest quartile of IgA in whey had a significantly lower relative risk for occurrence of AS (RR = 0.23; p = 0.02). In contrast, children of mothers with the high serum IgA (dichotomized) had 2.09 times higher relative risk (Figure 1; Table 4) compared to children of mothers with the low serum IgA. Infants exposed to the highest and the third quartile level of TGF-β1 in whey had a lower relative risk for occurrence of AS (RR = 0.3 and 0.26, respectively). Among the confounders, maternal history of eczema and household cigarette use at ages 6 and 12 months doubled the risk for AS (data not shown).

**Table 4:** Adjusted effects of immune markers in maternal serum and in whey on AS at ages 6 and 12 months

**Immune markers**^†^	***Immune markers in maternal serum before delivery***		***Immune markers in breast milk whey***	
	RR*	**p-value**	RR*	**p-value**
***Type-1/pro-inflammatory cytokines/chemokine****(pg/mL)*				
CXCL10				
4^th^ quartile (high)	2.30	0.01	1.31	0.46
3^rd^ quartile	1.57	0.13	0.43	0.03
2^nd^ quartile	0.96	0.90	0.68	0.28
***Type-2/pro-allergic cytokines/chemokines****(pg/mL)*				
IL-13				
4^th^ quartile (high)	3.02^‡^	0.004	4.18^‡^	0.001
3^rd^ quartile	2.20	0.03	1.12	0.13
2^nd^ quartile	1.10	0.78	1.91	0.83
IL-5				
4^th^ quartile (high)	4.34	0.001	2.24^‡^	0.01
3^rd^ quartile	1.85	0.18	n/a	n/a
2^nd^ quartile	2.27	0.05	n/a	n/a
***T-regulatory/anti-inflammatory cytokine****(pg/mL)*				
TGF-β1				
4^th^ quartile (high)	0.53	0.11	0.31^‡^	0.01
3^rd^ quartile	0.64	0.31	0.26	0.002
2^nd^ quartile	0.39	0.08	1.13	0.75
^***#***^***Immunoglobulin A****(mg/mL)*				
4^th^ quartile (high)	2.09	0.04	0.23^‡^	0.02
3^rd^ quartile	n/a	n/a	0.54	0.14
2^nd^ quartile	n/a	n/a	1.09	0.83

* Immune markers (except for IL-5 in whey and IgA in serum) were categorized into quartiles using the first quartile (lowest values) as reference. IL-5 in whey and IgA in serum were dichotomized. IL-5, IL-13, CXCL10, IgA, and TGF-β1 serum and whey were adjusted for child’s sex, maternal age during pregnancy, maternal race, smoking during pregnancy, vaginal infections during pregnancy, maternal history of asthma, eczema, and rhinitis, consumption of antibiotics during pregnancy, season of child’s birth, any respiratory infection during infancy, and household cigarette use at ages 6 and 12 months.

^#^ Secretory immunoglobulin A in whey but not in serum.

^†^ Gestational age, maternal education, pet exposure, and mode of delivery were removed from the models because they were not confounding.

^‡^ The overall effect of the quartiles was statistically significant after applying a false discovery rate (adjusted p value < 0.05).

Regarding the occurrence of asthma in the first six months and cumulative in the first 12 months, adjusted analyses of ever AS in infancy indicated that IL-5 and IL-13 in both maternal serum and whey, and TGF-β1 and IgA in whey had a significant influence on AS (supplemental material; Additional file 1: Table E3). In addition, IL-5, IL-13, IgA in both maternal serum and whey, and TGF-β1 in whey were associated with AS in the first 6 months of life (Additional file 1: Table E4).

We compared the associations between immune markers in maternal serum and whey with repeated measurements of AS at ages 6 and 12 months before and after statistically controlling for respiratory infections, wheezy bronchitis, and after excluding those who presented both wheezy bronchitis and AS. After controlling for wheezy bronchitis the RRs were similar to the adjusted risk ratios presented in Figure 1. Also after excluding children who had both wheezy bronchitis and AS, the RRs did not change. Hence, controlling for respiratory infections or excluding children with wheezy bronchitis did not essentially affect the associations of IL-5, IL-13, CXCL10, IgA, and TGF-β1 in both serum and whey with AS.

Finally, we determined whether specific combinations of low or high levels of IL-13 and IL-5 immune markers in serum or whey were associated with repeated AS in infants (Table 5). Compared to low levels of IL-13 in both serum and whey, low levels of IL-13 in serum and high levels in whey showed an increased relative risk for AS (IL-13: RR = 5.62). The combination of high levels of IL-5 in both serum and whey were marginally associated with AS compared to the low levels (RR = 2.13, p = 0.07).

**Table 5:** Adjusted effects of immune markers in maternal serum/breast milk whey on AS at ages 6 and 12 months

**Immune marker**^**†**^	aRR*	**p-value**
	**(95% CI)**	
**IL-13**		
High serum/high whey	1.62 (0.70, 3.75)	0.25
Low serum/high whey	5.62 (2.25, 14.03)	0.0002
Low serum/low whey	Reference	
**IL-5**		
High serum/high whey	2.13 (0.93, 4.88)	0.07
Low serum/high whey	0.77 (0.23, 2.57)	0.68
Low serum/low whey	Reference	

* IL-5 and IL-13 were adjusted for child’s sex, maternal age during pregnancy, maternal race, smoking during pregnancy, pelvic infections during pregnancy, maternal history of asthma, eczema, and rhinitis, consumption of antibiotics during pregnancy, season of child’s birth, any respiratory infection during infancy, and household cigarette use at ages 6 and 12 months.

^†^ Gestational age, maternal education, pet exposure, and mode of delivery were removed from the models because they were not confounding.

## Discussion

This is the first study to simultaneously examine the effect of immune markers in both maternal serum and breast milk whey on asthma-like symptoms in infants at ages 6 and 12 months. Results of the repeated measurement analyses suggest for individual immune markers that there is an increased risk of AS for higher IL-5 and IL-13 levels in maternal serum collected at the end of the pregnancy and whey. However, a combination of low levels of IL-13 in serum and high levels in whey increased the risk of AS. Higher individual concentrations of IgA and TGF-β1 in whey diminished the relative risk of AS, whereas higher IgA levels in maternal serum posed a risk.

With regards to selection bias, participation in studies during pregnancy and infancy depends on volunteering and a high level of dedication to study requirements. We enrolled 231 women and received breast milk or blood samples from 178 participants (77.1%). A strength of this study is the high compliance with clinical data collection since 78% (140/178) of the participants provided information on the child’s AS either at ages 6 or 12 months. We could not detect any association between the presence of AS symptoms and the likelihood of providing maternal blood/breast milk samples. However, our data show a potential selection. Based on the South Carolina Pregnancy Risk Assessment Monitoring System (PRAMS) survey data from 2006–2007, 21% of women obtained a graduate degree from college, whereas in our study 31% reported to graduate from college. It is known that women who smoked during pregnancy are more likely to be unmarried and have less than a high school education [30]. In our study 5% reported to smoke during pregnancy, whereas 16% of pregnant women in SC smoked during the last three months of pregnancy in 2008 [30]. However, since we adjusted for smoking during pregnancy in our explanatory models, the potential selection bias was diminished. In addition, maternal education did not confound the association between immune markers in both maternal serum and whey and asthma-like symptoms. Hence, the influence also of this potential selection bias is minimal.

Additionally, collecting duplicate measurements of each cytokine is likely to have improved the accuracy of the measurements. We also report similar findings for all cytokines measured in serum and milk whey. When comparable associations with AS are observed for cytokines obtained in both whey and serum samples, it is less likely that these findings are due to measurement errors or that they are due to chance. Hence, statistically significant findings for IL-5 and IL-13 in serum are corroborated by comparable results in whey and vice versa. Moreover, since levels of immune markers in whey may vary over time [31-34], we tested whether the immune mediators’ levels correlated with the interval of collection after birth. None of the immune markers were correlated with the time of milk collection, which is an agreement with a recent review on breast milk immune markers [35]. Regarding the dates of maternal blood sample collection, most immune markers except for IFN-γ were not correlated with the number of days of blood collection before delivery. Since IFN-γ was not associated with AS, there was no need to control for the dates of maternal blood sample collection in the other explanatory models.

Furthermore, interviews and collection of breast milk and venous blood and their chemical analyses were conducted independently. Therefore, there is no reason to assume that the information provided by the participants has distorted our results. In the statistical analyses we controlled for potential confounders. However, we did not control for breastfeeding duration since the immune markers are intervening variables between breastfeeding and asthma-like symptoms (a chain of responses). It is not appropriate to split a chain of responses up into their elements and assess them individually since the elements of one chain are dependent on one another. The product of breastfeeding duration (median: 4 weeks) and immune markers in breast milk was also not considered given that such a product will produce large uncertainty since immune markers were only measured once and their level may change during the course of breastfeeding [35]. It is possible that other unmeasured confounders may predispose the child to develop asthma-like symptoms in infancy such as the exposure to house dust mites, area of residence, day care attendance, and parity. Genetic susceptibility, environmental factors, and geographical location of the host may explain the discrepancies found between our study and others [36,37]. Finally, regarding the sample size, this study is one of the larger investigations. Only five studies reported so far on immune markers in whey had sample sizes of 100 or more [35].

To estimate the effect of infections on AS, we conducted four alternate approaches: (1) not excluding children with AS and wheezy bronchitis; (2) using wheezy bronchitis as a confounder; (3) using the four respiratory infections variable as one confounder, and (4) excluding all cases with wheezy bronchitis. The results of these approaches showed similar associations; hence a misclassification of asthma-like symptoms as respiratory infections is unlikely to explain our findings. There is no consensus in the literature as to what AS in childhood represents [38,39]. It is generally considered that AS early in life are related to respiratory viruses [39], but not allergies. However, these symptoms in children, although related to viruses initially, may become allergic [39]. Hence, AS in infancy are more likely to reflect an unspecific response of the respiratory system to external antigenic challenges in general (*locus minoris resistentiae*), rather than a specific response to a specific type of antigenic challenge.

Another limitation of our study is that we ran separate models for each immune marker. The drawback of this approach is that the effect of each cytokine was not adjusted for other proteins in serum and whey. However, the adjustment for other proteins would have presented collinearity issues, which would have biased the risk estimates. One way to address this issue is to determine latent patterns or factors that incorporate multiple immune markers. However, this approach will not provide specific information for the various markers. After adjusting for multiple testing using the false discovery rate, the statistical effects of IL-13 in serum, and IL-5, IL-13, IgA, and TGF-β1 in whey remained significant (Table 4).

Regarding the median concentration of immune markers in maternal serum, IFN-γ, IgA, and TGF-β1 were similar to other maternal serum studies [35], but IL-5, IL-6, and IL-13 were lower. Regarding the concentrations of the immune markers detected in whey, the median of IgA, TGF-β1, CXCL10, and IFN-γ were comparable to other breast milk studies recently reviewed by Agarwal et al. [35]. The other cytokines (IL-5, IL-6, and IL-13) and chemokine (CXCL8) levels in whey were lower, although they still fell within the range reported by other studies. Previous studies have reported that TGF-β1 measurement in plasma is preferable compared to serum because a major part of this marker is released by platelets during clotting [40]. However, for complete release of TGF-β1 from serum, we incubated serum overnight at 2 - 8°C before centrifugation and then followed the activation manufacture procedure [24].

### Type-2 cytokines

Our results suggest that two Type-2 markers (IL-5 and IL-13) were risk markers for the occurrence of AS in infants. It is known that Th2 cells orchestrate allergic inflammation through the release of the Type-2 cytokines IL-5 and IL-13 [41]. In particular, IL-5 has been linked to eosinophil-mediated inflammatory response, IL-4 and IL-13 in the isotype switching of B-cells to IgE production and IL-13 is a critical player in increased mucus secretion [42-44]. Thus, IL-13 and IL-5 cytokines are considered to play a prominent role in the pathophysiology of asthma [45]. This is the first study to demonstrate a link of IL-5 and IL-13 in maternal serum and milk whey with AS in infants.

### Type-1 cytokines/chemokines

The Type-1 cytokines are responsible for cell-mediated immunity and phagocytes-dependent protective responses. Cytokines/chemokines produced by Th1 cells include IFN-γ, IL-12(p70), and CXCL10.

Our results suggest that the exposure to high levels of CXCL10 (IP-10) during gestation may predispose the fetus to asthma-like symptoms in infancy, however the third quartile level of this cytokine was protective. To date, no studies have looked at the effect of this marker in maternal serum and in whey on asthma-like symptoms in infants, creating the necessity for further assessment. In an additional analysis, we explored whether an imbalance of Th1 and Th2 immune markers (ratios of Th2 to Th1 cytokines) affects the occurrence of AS in infancy. No association of any ratio with AS was found.

### T-regulatory/anti-inflammatory cytokines and IgA

We identified that the highest quartiles of TGF-β1 and IgA in whey were related to a 69% and 77% diminished probability of AS, respectively. Some previous studies reported that differences in secretory immunoglobulin A (sIgA) in breast milk did not affect the development of childhood allergies [46-49]. However, other studies have demonstrated that higher levels of sIgA [50] and TGF-β1 [51] in breast milk are protective against the development of allergy [52,53], and wheezing during infancy [54]. Our finding on TGF-β1 is in agreement with the results reported by Oddy et al., since they found that the risk of wheeze was lower when TGF-β1 was increased (long breastfeeding and medium-high TGF-β1 levels compared with short breastfeeding and low TGF-β1 level).

Interestingly, higher concentrations of IgA (geometric mean: 12.88 mg/ml) in whey than in serum (geometric mean: 4.73 mg/ml, Table 3) suggest that whey IgA is locally derived from the mammary glands [35]. The negative correlation found between IgA in serum and whey, may be due to the fact that the predominant IgA isotype presented in serum is IgA1, whereas in whey it is predominantly IgA2 [55,56]. A limitation is that our ELISA kit did not distinguish these two isotypes. Moreover, the source of circulating IgA in predominantly bone marrow plasma cells while the source of secretory IgA is from mucosal cells [57].

It is known that maternal IgA does not cross the placenta and its levels are low in cord blood [58]. Hence, it was surprising to find that higher maternal serum IgA (probably reflecting higher IgA1) posed an increased risk for asthma-like symptoms in the infant. We do not know how to explain this association. To the best of our knowledge, no previous study has found such association, and therefore, this finding needs further evaluation. During early infancy, the baby’s intestinal production of IgA is low [59]. It is considered that the maternal IgA provides the protection against environmental pathogens. Maternal IgA seems to stimulate the offspring production of IgA, preventing the child from developing allergies [60].

In this study, both maternal serum and whey immune markers (IL-13 and IL-5) increased the risk of AS in the offspring from South Carolina. Given that immune markers in maternal serum and whey are correlated (Table 3), the results suggests that the child had a similar exposure to immune markers in late gestation (0 to 13 weeks before delivery) and postnatal period. This brings us to the question of whether exposure during late gestation or after birth is more influential for AS. It is believed that antenatal events (including dietary nutrients, microbial products, and cigarette smoking) may play a role in the development of allergic diseases [61]. Prescott et al. found that the capacity of the fetus to produce IL-13 and IL-10 was directly related to the levels of these cytokines produced by the mother in response to fetal antigens, implying that the level of immune reactivity at the feto-maternal interface may influence the level and pattern of evolving fetal immune responses [61]. If there is passage across the placenta, the infant’s contact with specific immune markers in breast milk is likely not to be a *de novo* exposure [36]. Our results propose that having the combination of low IL-13 in serum and high in whey was significantly associated to AS, signifying that high levels of this marker in whey pose a risk for AS. Since gestational and postnatal exposures to immune factors may contribute to an infant’s risk of developing immune-mediated disorders [62], it is important to decipher the role of both prenatal and post-natal immune factors in the development of respiratory immune responses of the infant.

## Conclusion

In summary, this is the first longitudinal study to investigate the effect of immune markers in maternal serum before delivery and in whey on the occurrence of AS in infants. Our study is one of few with a sample size of 100 and more [36]; and it seems that both maternal serum and whey provide a multitude of immune markers that affect the occurrence of AS in infants. Our results support previous findings showing a protective effect of IgA and TGF-β1 against the occurrence of AS in children. Although IL-5 and IL-13 are known to be involved in the pathogenesis of asthma, no breast milk study has demonstrated associations with AS previously. Therefore, we were surprised to find that these cytokines in serum and whey were strong risk markers. These effects warrant further investigations. When only breast milk is investigated, it seems as if milk cytokines IL-5 and IL-13 have adverse effects. However, similar immune exposure during gestation and via milk suggests that both increase AS among infants. Consequently, future studies need to take maternal immune markers in serum into consideration when assessing the risk of immune markers in breast milk.

## Abbreviations

AS: Asthma-like symptoms; IL: Interleukins; Th: T helper; Ig: Immunoglobulin; IFN: Interferon; TGF: Transforming growth factor; CXCL10: IFN gamma-induced protein 10; ELISA: Enzyme-linked immunosorbent assay; LOD: Limit of detection; ISAAC: International study of asthma and allergies in childhood; RR: Risk ratio; CI: Confidence interval; min: Minutes; pg: Pictograms; mg: Milligrams; mL: Milliliters; rpm: Revolutions per minute; °C: Degrees celsius.

## Competing interests

The authors of this study have no competing interest to declare.

## Authors’ contributions

NSR carried out the immunoassays and the phone interviews, performed the statistical analysis, and drafted the manuscript. WK developed the study, and participated in its design and coordination and helped to draft the manuscript. MY helped to perform the immunoassays and phone interviews. HZ participated in the statistical analysis and helped editing the manuscript. JL helped to draft the manuscript. VG helped with the interpretation of the data and to draft the manuscript. All authors read and approved the final manuscript.

## Authors’ information

NSR was a doctoral student from the Epidemiology and Biostatistics department at the University of South Carolina. NSR has a Master in Public Health and in Microbiology. NSR’s research experience has been focused on maternal and child health, in particular allergy, asthma, and eczema in children. NSR’s interest is to understand how the child immune system is influenced by early life exposures, including breastfeeding duration, immune markers in maternal serum and breast milk whey.

WK has been a professor in the Department of Epidemiology and Biostatistics at the University of South Carolina since 2005. Previously, WK was an associate professor in the Department of Epidemiology at Michigan State University. WK’s interest covers environmental exposures and health outcomes in the life span from pre-conception to adolescence (maternal and child health). The overarching objective of his research is to disentangle the effects of pre- and postnatal exposures, which ultimately would lead to improved public health policies.

MY is a Doctoral student from the Epidemiology department at the University of South Carolina. MY’s research interests focus on prenatal, early life, and adolescent exposures as they relate to lung function and asthma in early adulthood.

HZ is an Assistant Professor of Biostatistics. Her research interests focus on developing Bayesian methods applied to biological area, including progeny identification using genetic data, clustering methods, multi-level causes of a certain type of disease, measurement error modeling, and variable selections. Since 2007, HZ has worked with WK on various projects related to genetic polymorphisms related to atopy, asthma, and eczema, epigenetic effects, gene expression analysis, and heterosis.

JL is an Associate Professor of Epidemiology at the University of South Carolina. Her interests focus on perinatal epidemiology, reproductive health, social and demographic determinants of health, survey data collection and analysis, and international health.

VG is an Associate Professor of Immunology at Michigan State University. VG’s research interests cover food allergens, food allergy, anaphylaxis, asthma, immunology, breast milk immunology, assessment of allergenicity of food using mouse model of food allergy, and dietary modification to prevent/treat food allergy.

## Supplementary Material

Additional file 1 **Table E1.** Spearman correlations between immune markers in maternal serum during gestation. **Table E2.** Spearman correlations between immune markers in breast milk whey. **Table E3.** Adjusted effects of immune markers in maternal serum and in whey on ever AS in the first year of life. **Table E4.** Adjusted effects of immune markers in maternal serum and in whey on AS at age 6 months. Click here for file
