# Supplementary material for: Maternal immune markers in serum during gestation and in breast milk and the risk of asthma-like symptoms at ages 6 and 12 months: a longitudinal study
Source: Allergy Asthma Clin Immunol. 2012 Jul 17;8(1):11. doi: 10.1186/1710-1492-8-11 (PMC3536636; doi:10.1186/1710-1492-8-11)
Supplement: Additional file 1 — Table E1. Spearman correlations between immune markers in maternal serum during gestation. Table E2. Spearman correlations between immune markers in breast milk whey. Table E3. Adjusted effects of immune markers in maternal serum and in whey on ever AS in the first year of life. Table E4. Adjusted effects of immune markers in maternal serum and in whey on AS at age 6 months. [file 1710-1492-8-11-S1.pdf]

Table E1. Spearman correlations between immune markers in maternal serum during gestation

	Immune markers in maternal serum							
	IFN-γ	IL-13	IL-5	IL-6	CXCL10	IL-8	IgA	TGF-β1
	(In the following order: correlation coefficient, p-value, n)							
IFN-γ	1	0.37	0.36	0.50	0.12	0.10	0.03	-0.21
		<.0001	<.0001	<.0001	0.12	0.25	0.76	0.04
		161	128	128	161	128	85	84
IL-13		1	0.77	0.71	0.44	0.41	0.14	-0.16
			<.0001	<.0001	<.0001	<.0001	0.17	0.13
			128	128	161	128	85	84
IL-5			1	0.80	0.54	0.36	0.11	-0.10
				<.0001	<.0001	<.0001	0.31	0.38
				128	128	128	74	71
IL-6				1	0.53	0.42	0.17	-0.06
					<.0001	<.0001	0.13	0.57
					128	128	74	71
CXCL10					1	0.45	0.09	0.01
						<.0001	0.38	0.88
						128	85	84
IL-8						1	0.15	0.12
							0.18	0.29
							74	71
IgA							1	-0.11
								0.29
								82
TGF-β1								1

Table E2. Spearman correlations between immune markers in breast milk whey

	Immune markers in whey							
	IFN-γ	IL-13	IL-5	IL-6	CXCL10	IL-8	IgA	TGF-β1
	(In the following order: correlation coefficient, p-value, n)							
IFN-γ	1	0.47	0.05	0.13	-0.04	0.06	-0.01	-0.16
		<.0001	0.60	0.23	0.64	0.57	0.89	0.12
		115	84	84	115	84	89	86
IL-13		1	0.09	0.05	0.10	0.40	-0.21	-0.28
			0.37	0.64	0.26	0.0002	0.03	0.008
			84	84	115	84	89	86
IL-5			1	0.31	-0.17	0.01	0.06	0.07
				0.004	0.10	0.89	0.58	0.52
				84	84	84	73	71

IL-6	1	0.36	0.52	0.23	0.44
		0.0007	<.0001	0.04	<.0001
		84	84	73	71
CXCL10		1	0.68	0.04	0.20
			<.0001	0.70	0.06
			84	89	86
IL-8			1	0	0.18
				1	0.12
				73	71
IgA				1	0.30
					0.004
					85
TGF-β1					1

Table E3. Adjusted effects of immune markers in maternal serum and in whey on ever AS in the first year of life

**Immune markers**†	***Immune markers in maternal serum before delivery***		***Immune markers in breast milk whey***	
	**RR**	**p-value**	**RR**	**p-value**
Type-2/pro-allergic cytokines/chemokines *(pg/mL)*				
IL-13				
4th quartile (high)	3.04	0.001	3.66	0.001
3rd quartile	2.72	0.003	2.69	0.01
2nd quartile	1.07	0.84	1.00	0.99
IL-5				
4th quartile (high)	4.44	0.0004	2.53	0.004
3rd quartile	2.38	0.05	n/a	n/a
2nd quartile	2.29	0.07	n/a	n/a
T-regulatory/anti-inflammatory cytokine *(pg/mL)*				
TGF-β1				
4th quartile (high)	0.55	0.19	0.41	0.04
3rd quartile	0.56	0.14	0.32	0.008
2nd quartile	0.32	0.03	1.17	0.66
#***Immunoglobulin A*** *(mg/mL)*				
4th quartile (high)	1.68	0.15	0.34	0.04
3rd quartile	n/a	n/a	0.77	0.55
2nd quartile	n/a	n/a	1.36	0.44

†Immune markers (except for IL-5 in whey and IgA in serum) were categorized into quartiles using the first quartile (lowest values) as reference. IL-5 in whey and IgA in serum were dichotomized. IL-5, IL-13, CXCL10, IgA, and TGF-β1 serum and whey were adjusted for child's sex, maternal age during pregnancy, maternal race, smoking during pregnancy, pelvic

infections during pregnancy, maternal history of asthma, eczema, and rhinitis, consumption of antibiotics during pregnancy, season of child's birth, any respiratory infection during infancy, and household cigarette use.
[#] Secretory immunoglobulin A in whey but not in serum

Table E4. Adjusted effects of immune markers in maternal serum and in whey on AS at age 6 months

**Immune markers**[†]	***Immune markers in maternal serum before delivery***		***Immune markers in breast milk whey***	
	**RR**	**p-value**	**RR**	**p-value**
***Type-2/pro-allergic cytokines/chemokines** (pg/mL)*				
IL-13				
4th quartile (high)	3.96	0.0004	12.84	0.001
3rd quartile	2.44	0.02	5.01	0.05
2nd quartile	0.66	0.38	2.33	0.32
IL-5				
4th quartile (high)	6.50	0.0002	2.49[‡]	0.03
3rd quartile	2.56	0.56	n/a	n/a
2nd quartile	1.42	0.06	n/a	n/a
***T-regulatory/anti-inflammatory cytokine** (pg/mL)*				
TGF-β1				
4th quartile (high)	0.65	0.45	0.26	0.009
3rd quartile	0.77	0.61	n/a	n/a
2nd quartile	0.51	0.26	n/a	n/a
***[#]Immunoglobulin A** (mg/mL)*				
High levels	3.05	0.01	0.24	0.003

[†]Immune markers (except for IL-5, IgA in whey and in serum and TGF-β1 in whey) were categorized into quartiles using the first quartile (lowest values) as reference. IL-5, IgA in whey and in serum and TGF-β1 in whey were dichotomized. IL-5, IL-13, CXCL10, IgA, and TGF-β1 serum and whey were adjusted for child's sex, maternal age during pregnancy, maternal race, smoking during pregnancy, pelvic infections during pregnancy, maternal history of asthma, eczema, and rhinitis, consumption of antibiotics during pregnancy, season of child's birth, any respiratory infection during the first 6 months of life, and household cigarette use.
[#] Secretory immunoglobulin A in whey but not in serum
